# Peptide-Pulsed Dendritic Cells Have Superior Ability to Induce Immune-Mediated Tissue Destruction Compared to Peptide with Adjuvant

**DOI:** 10.1371/journal.pone.0092380

**Published:** 2014-03-19

**Authors:** Dilan Dissanayake, Kiichi Murakami, Michael D. Tran, Alisha R. Elford, Douglas G. Millar, Pamela S. Ohashi

**Affiliations:** 1 Campbell Family Institute for Breast Cancer Research, Princess Margaret Cancer Center, University Health Network, Toronto, Ontario, Canada; 2 Department of Immunology, University of Toronto, Toronto, Ontario, Canada; Maisonneuve-Rosemont Hospital, Canada

## Abstract

Vaccines for cancer immunotherapy are of interest but in general have not yet achieved the desired therapeutic efficacy in clinical trials. We present here a novel model to evaluate vaccine strategies by following tissue destruction in a transgenic model, where a defined antigen is expressed on pancreatic islets. We found that the transfer of syngeneic antigen-pulsed dendritic cells (DCs) resulted in autoimmune cytotoxic T-lymphocyte activation that was not observed following vaccinations that were based on peptides and adjuvants. Importantly, the induction of diabetes by DC transfer is dependent upon the maturation of DCs prior to transfer. Furthermore, diabetes induction only occurred if DCs were pulsed with the immunodominant epitope in addition to at least one other peptide, suggesting greater cytolytic activity upon engagement of multiple T-cell specificities. While the tumor environment undoubtedly will be more complex than healthy tissue, the insights gained through this model provide useful information on variables that can affect CD8-mediated tissue cytolysis *in vivo*.

## Introduction

Numerous studies have described the existence of tumor-specific CD8^+^ T-cells in the periphery of cancer patients that have neither been deleted nor functionally inactivated [1], [2], [3]. A goal of cancer vaccination is to properly activate these cells to promote the lysis of tumor tissue. While a great deal of progress has been made in the identification of tumor-associated antigens, therapeutic responses have been limited and much work remains to be done in order to achieve long-term clinical remission using these techniques [4], [5], [6]. Two specific methods of vaccination being used in clinical trials involve the administration of either tumor-associated antigenic peptides alongside adjuvants [7], [8], [9], or the *ex vivo* generation, maturation and loading of autologous dendritic cells (DCs) with tumor antigens, which are then transferred to the patient [10], [11], [12]. While peptide vaccines and DC vaccines have both been shown to successfully promote varying degrees of expansion, cytokine production, and cytotoxic T-lymphocyte (CTL) activity, it is currently unclear whether either strategy is superior at eliciting tissue destruction. Furthermore, failures in tumor vaccination approaches have been attributed to the inherent heterogeneity and instability of tumors as a target tissue, as well as the various modes of immune evasion exhibited by tumors such as immune editing, induction and recruitment of regulatory T-cells (Tregs), expression of inhibitory molecules, and local vascular alterations [13], [14], [15], [16]. Effective immunotherapy will require not only an understanding of these immunosuppressive networks but also better evaluation of various immunization strategies for their effectiveness at properly inducing T-cells that are capable of tissue cytolysis.

Since many autoimmune disorders, including type I diabetes and multiple sclerosis, are characterized by the activation of T-cells against self-tissue, models of autoimmunity may provide useful insights regarding the parameters that must be fulfilled to initiate T-cell mediated tissue pathology. Several models have been used to explore the factors that contribute to autoimmunity. For example, the selective deletion of FoxP3^+^ Tregs has been shown to result in T-cell activation and systemic autoimmune disease [17], [18]. Alternatively, a number of groups have used a CD4^+^ adoptive transfer model to examine Treg suppressive function in experimental colitis [19]. The experimental autoimmune encephalitis model has also been used extensively to explore factors involved in the activation of CD4^+^ T-cells against antigens of the central nervous system [20]. Our lab has used a mouse (RIP-gp) that possesses a transgene for a glycoprotein (gp) derived from the lymphocytic choriomeningitis virus (LCMV) under the control of the rat insulin promoter (RIP). This allows specific expression of gp in the insulin-producing β cells of the pancreas, where it can serve as a model antigen towards which CD8^+^ anti-tissue responses can be readily measured [21]. It has been shown that T-cells specific towards gp remain ignorant of its presence in the pancreas unless properly activated, by infection with LCMV for example, which leads to infiltration and destruction of the β cells and consequent diabetes. Importantly, in this context the gp antigen does not act as a viral signal as evidenced by the fact that RIP-gp mice do not display signs of constitutive immune activation or inflammation [21]. Furthermore, gp has previously been used as a useful model antigen for exploring immune responses against tumors, and has behaved similarly to human tumor-associated antigens by eliciting measurable but limited T-cell mediated anti-tumor activity [22], [23], [24], [25].

Here, we use vaccination techniques in the RIP-gp mouse model to explore the requirements for the activation of tissue-specific T-cells, with the goal of identifying strategies that lead to the effective induction of tissue destruction. Using this model, we compared vaccination protocols that rely on the administration of peptides and adjuvant to those relying on the transfer of antigen-bearing DCs, for their effectiveness at eliciting CTL-mediated islet cytolysis. We found that, even with significant expansion of antigen-specific T-cells and infiltration of the pancreatic islets, peptide vaccination is unable to induce diabetes in the RIP-gp mouse. With DC vaccination on the other hand, mice become hyperglycemic in a manner that is dependent upon the maturation status of the transferred DCs and the engagement of multiple T-cell populations specific for gp-derived epitopes. This work suggests that DC vaccines may elicit more effective CTL activity than vaccines consisting of peptides and adjuvant, and that polyclonal T-cell activation may be a requirement for robust tissue cytolysis.

## Materials and Methods

### Mice and Diabetes Monitoring

All mice were maintained in a specific-pathogen-free facility. This work was approved by the Ontario Cancer Institute Animal Resource Centre in accordance with institutional guidelines and the guidelines of the Canadian Council on Animal Care. The C57BL/6 mice used in the studies were purchased from Jackson Laboratories (Bar Harbor, ME).

The RIP-gp mouse line has been described previously [21], as have the P14 and Smarta TCR transgenic mice [26], [27]. Congenic Thy1.1^+^ mice were purchased from Jackson Laboratories (*B6.PL-Thy1^a^/CyJ*) and crossed to the P14 and Smarta transgenic mice. Blood glucose measurements were taken 2–3 times per week using Accu-chek III Glucometers and Chemstrips (Roche) and mice were considered diabetic following 2 consecutive measurements of >14 mM.

### Reagents and Antibodies

Splenocyte harvests and T-cell cultures were conducted in Iscove’s media containing 10% heat inactivated fetal calf serum (PAA Laboratories), 50 μM 2-mercaptoethanol (Sigma), 2 mM L-glutamine (Invitrogen) and antibiotics.

DC cultures and co-cultures were conducted in RPMI 1640 containing 10% LPS-free fetal calf serum (Gibco), 50 μM 2-mercaptoethanol (Sigma), 2 mM L-glutamine (Invitrogen) and antibiotics. GM-CSF was purchased from Peprotech.

Peptides were purchased from Washington Biotechnology (gp33–41 (KAVYNFATM), gp276–286 (SGVENPGGYCL) and adenoviral (AV) peptide (SGPSNTPPEI)) and New England Biolabs (gp61–80 (GLNGPDIYKGVYQFKSVEFD)).

Antibodies specific for CD8, CD4, and TNF-α were purchased from BD Pharmingen and antibodies specific for Thy1.1, IFN-γ, and IL-2 were purchased from eBioscience. Carboxyfluorescein succinimidyl ester (CFSE) was purchased from Molecular Probes.

### Peptide Vaccination

Mice were infused intravenously via tail vein on days 0 and 2 with 10 μg gp33–41, 10 μg gp276–286, and 2 μg gp61–80 peptides, with or without 50 μg CpG ODN 1826 (ACGT), 30 μg LPS (Sigma), or 2 mg anti-CD40 agonistic antibody FGK45 [28] as indicated in Hanks Buffered Salt Solution (HBSS) with a total volume of 200 μl per injection.

### Tetramer Staining

Tetramers were prepared from monomers for H-2D^b^:KAVYNFATM and H-2K^b^:AVYNFATC (Baylor) and fluorochrome-conjugated extravidin (Invitrogen). Tetramer staining was conducted by incubating for 30–60 minutes at 4 degrees Celsius prior to surface staining for other molecules.

For tetramer staining of blood, lymphocytes were enriched prior to staining using the following protocol. Blood was collected in tubes containing 4% sodium citrate, which were filled with complete Iscove’s medium. Cells were then underlayed with Histopaque 1077 (Sigma) and centrifuged at 2000 r.p.m. for 20 minutes at room temperature. The buffy coat was transferred to new tubes containing fresh medium and washed once prior to staining with tetramers and surface antibodies.

For tetramer staining of splenocytes, spleens were harvested and processed to single cell suspensions. Red blood cells were lysed with ammonium chloride lysis buffer prior to tetramer staining.

### Virus Infection

Mice were infected with 2500 p.f.u. of LCMV-Armstrong strain via tail vein infusion.

### Pancreatic Immunohistochemistry

Freshly removed pancreases were placed in cryomolds (Tissue-Tek) and immersed in O.C.T. compound (Tissue-Tek) prior to snap-freezing in liquid nitrogen and storage at −80 degrees Celsius. Frozen tissue sections of 5 μm thickness were cut in a cryostat, placed on siliconized glass slides, air-dried and fixed in acetone for 10 minutes. Rehydrated tissue sections were stained with primary antibodies followed by alkaline phosphatase-linked anti-Igs. Alkaline phosphatase was visualized using naphthol AS-BI phosphate and new fuchsin as substrate, yielding a red color reaction product. Endogenous alkaline phosphatase was blocked by levamisole. Quantification was performed using light microscopy.

### Dendritic Cell Generation and Transfer

DCs were generated as previously described [29]. Briefly, bone marrow harvested from mouse femurs and tibiae was cultured in 10 mls of complete RPMI containing 40 ng/ml GM-CSF (Peprotech) in bacteriological (non-tissue culture treated) plates (BD Falcon). On day 3, an additional 10 mls of complete RPMI containing 40 ng/ml GM-CSF was added. On days 6 and 8, 10 mls was removed from the plate, centrifuged at 1400 r.p.m. for 5 min. and cell pellets were resuspended in fresh GM-CSF-containing media and added back to plates. On day 9 or 10, nonadherent DC were harvested and cultured with CpG ODN 1826 (10 μM), LPS (10 ng/ml), or imiquimod acetate (Sequoia) (25 μM) at 2×10^6^ cells/well in tissue-culture treated 24 well plates for 16–20 hours. For transfer, DCs were pulsed for 2 hrs with 1 μg/ml each of gp33–41, gp276–286, and gp61–80. Cells were then harvested from plates and washed two to three times in HBSS prior to intravenous administration at 2×10^6^ cells per mouse through tail vein infusion.

### Magnetic Cell Separation

Magnetic-activated Cell Sorting (MACS) was conducted using reagents from Miltenyi Biotec according to manufacturer’s instructions. Briefly, spleens were harvested and processed to single-cell suspension. Splenocytes were resuspended without red blood cell lysis in autoMACS running buffer and magnetic labeling was conducted using the CD8^+^ T-cell Isolation Kit II or CD4^+^ T-cell Isolation Kit II. Negative selection was performed using LS MACS separation columns to enrich for CD8^+^ or CD4^+^ cells as indicated.

### *In vivo* Proliferation Assay

Spleens from P14 or Smarta transgenic mice were harvested and processed to single cell suspensions. CD8^+^ or CD4^+^ cells were enriched by magnetic separation as described above. CFSE labeling was conducted by incubating in FCS-free media with 10 μM CFSE for 10 min at 37 degrees Celsius. 2×10^6^ CFSE-labeled cells were transferred intravenously by tail vein infusion. Splenocytes were harvested 3 days later and stained for CD8 or CD4 as appropriate, and Thy1.1 prior to flow cytometry for CFSE dilution. For mice receiving co-transfer of DCs, T-cell and DC transfers were conducted as two separate infusions with the second occurring in the contralateral vein to the first. In all experiments, CFSE-labeled cells were also transferred to naïve animals to determine fluorescence intensity of undivided cells.

### *In vivo* Cytotoxicity Assay

Cytotoxicity assays were performed as described previously [30]. Briefly, splenocytes from C57Bl/6 mice were pulsed with 1 μg/ml gp33–41 or AV control peptides for 2 hrs at 37 C, washed, and labeled with high dose (10 μM) or low dose (1 μM) CFSE, respectively. Equal numbers of the labeled target cells (1.5×10^7^) were transferred intravenously into mice on day 6 post vaccination. 4 hrs later, total splenocytes were isolated and examined for specific target lysis by flow cytometry. % lysis was calculated as (% AV-labeled cells - %-gp33–labeled cells)/(% AV-labeled cells)×100.

### Flow Cytometry

Surface staining of markers was conducted at 4 degrees Celsius for 30 min. followed by 2 washes prior to fluorescence detection. Samples were acquired on FACSCalibur instruments (BD) and were analyzed using FlowJo software (Tree Star).

Intracellular staining was performed by incubating cells in Cytofix/Cytoperm (BD Pharmingen) after surface staining for 20 min. at 4 degrees Celsius, followed by incubation with mAbs diluted in 1× Perm/Wash Buffer (BD Pharmingen) for 30 min. at 4 degrees Celsius. For intracellular cytokine detection, cells were stimulated in the presence of GolgiPlug (BD Pharmingen) for 5 to 6 hours prior to fixation and permeabilization.

### *In vivo* CD4 and CD8 Depletion

CD4 and CD8 depletion of mice was conducted by intravenous infusion via tail vein of YTS191 and YTS169 antibodies on days −3 and −1 prior to DC vaccination.

### Statistical Analysis

Significant differences between groups were determined using Student’s t-test. Differences between Kaplan-Meier plots were determined by log-rank test using GraphPad Prism software.

## Results

### Peptide Vaccinations with Adjuvant are Insufficient to Induce Diabetes in RIP-gp Mice

In order to evaluate the efficacy of tissue destruction induced by peptide vaccination, RIP-gp mice were vaccinated by intravenous infusion on day 0 and day 2 with a peptide cocktail consisting of the MHC class I restricted immunodominant gp33–41 and subdominant gp276–286 epitopes [31], and the MHC class II restricted gp61–80 epitope [32]. As summarized in Table 1, peptide vaccination alone or in conjunction with the administration of CpG ODN 1826, LPS, or anti-CD40 agonist antibody did not result in hyperglycemia in RIP-gp single-transgenic mice. As a positive control, double transgenic P14/RIP-gp mice that include a biased T-cell receptor (TCR) repertoire, which is specific for the gp33–41 peptide in the context of MHC H-2D^b^, were immunized with similar peptide cocktails. Similar to previous results using P14/RIP-gp mice [33], [34] or the co-transfer of large numbers of transgenic T-cells [35], diabetes was only induced upon transfer of gp peptides together with agents that promoted antigen-presenting cell (APC) maturation, and not with peptide alone. Notably, the anti-CD40 agonistic antibody was superior at inducing diabetes, followed by CpG and LPS (Table 1). This demonstrated that the immunization strategies were capable of activating T-cells *in vivo* with a heavily biased T-cell repertoire specific for the antigen-bearing islets, yet were insufficient to induce optimal T-cell responses in a normal repertoire.

**Table 1:** Induction of diabetes in P14/RIP-gp and RIP-gp mice using peptide/adjuvant vaccines.

Mouse Strain	Peptides (day 0 and day 2 i.v.)	Adjuvant (day 0 and day 2 i.v.)	% of diabetic mice
P14/RIP-gp	10μg gp33–41+10μg gp276–286+2μg gp61–80	None	0%
RIP-gp	10μg gp33–41+10μg gp276–286+2μg gp61–80	None	0%
P14/RIP-gp	10μg gp33–41+10μg gp276–286+2μg gp61–80	30 μg LPS	50%
P14/RIP-gp	10μg gp33–41+10μg gp276–286+2μg gp61–80	50 μg CpG ODN 1826	80%
P14/RIP-gp	10μg gp33–41+10μg gp276–286+2μg gp61–80	Anti-CD40 agonist antibody	100%
RIP-gp	10 μg gp33–41+10μg gp276–286+2μg gp61–80	30 μg LPS	0%
RIP-gp	10 μg gp33–41+10μg gp276–286+2μg gp61–80	50 μg CpG ODN 1826	0%
RIP-gp	10 μg gp33–41+10μg gp276–286+2μg gp61–80	Anti-CD40 agonist antibody	0%

Mice were infused intravenously with indicated peptides and adjuvants on days 0 and 2 and blood was drawn every 2–3 days from the tail vein to assess blood glucose levels for a minimum of 16 days. Diabetes is defined as a blood glucose of >14 mM on two consecutive readings. Indicated results are for at least 5 mice per group and 2–3 independent experiments.

### Combination of Anti-CD40 agonist Antibody and TLR Ligand does not Induce Diabetes in RIP-gp Mice

**Figure 1:**
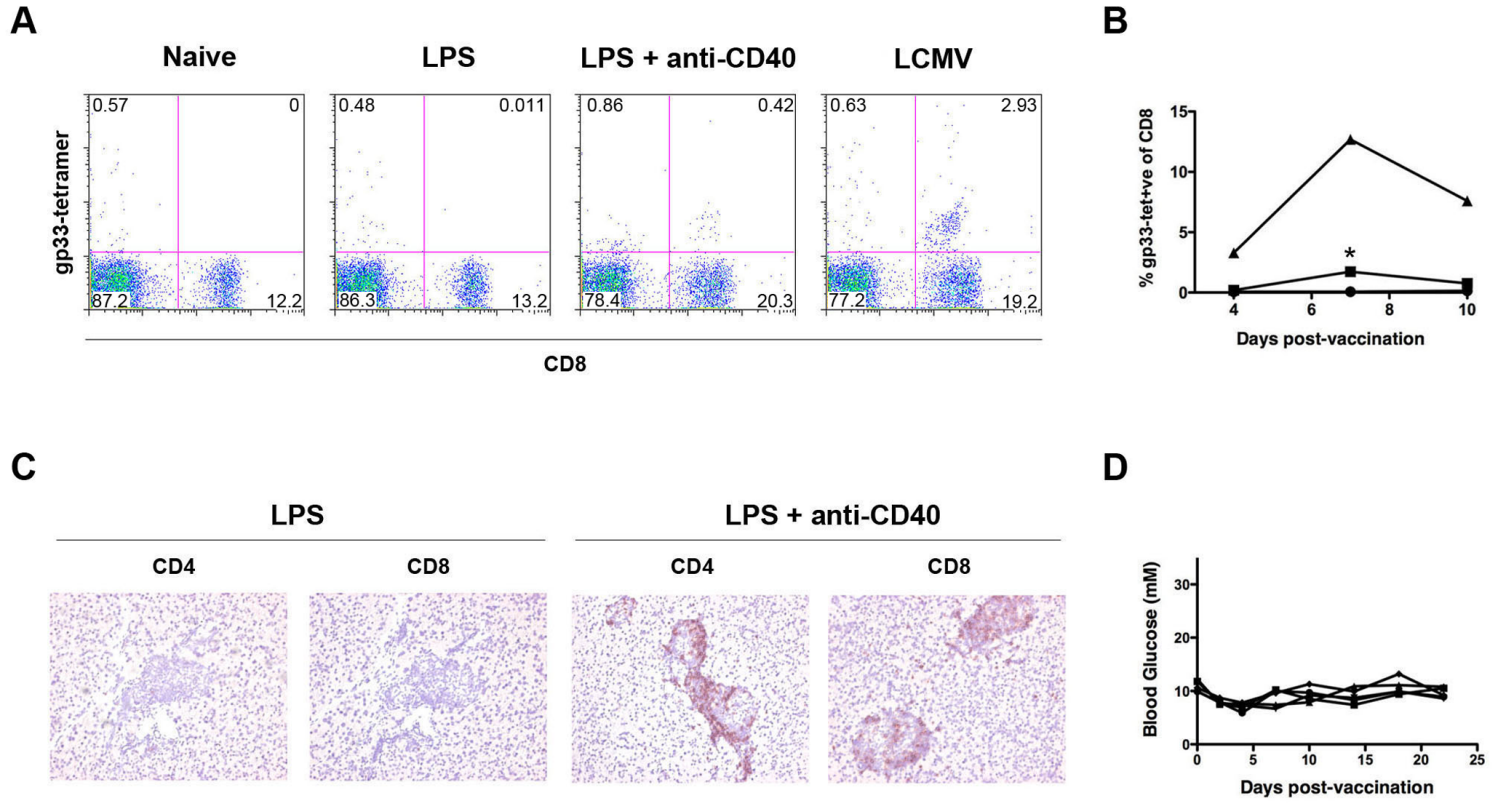
Peptide vaccination with anti-CD40 and LPS promotes CD8^+^ cell expansion and infiltration but not diabetes. Mice were infused intravenously on days 0 and 2 with 10 μg gp33–41, 10 μg gp276–286, 2 μg gp61–80, and 30 μg LPS with or without anti-CD40 agonistic antibody as indicated. (A) Flow cytometry conducted on blood of C57BL/6 mice 7 days after the first vaccination stained using anti-CD8 antibody and gp33–41 tetramer. Representative plots are shown. Naïve and LCMV-infected mice were used as negative and positive controls respectively. (B) Quantification of tetramer-positive populations in blood of C57BL/6 mice after infection with LCMV (triangles) or vaccination with peptides in conjunction with LPS (circles), or LPS and anti-CD40 (squares). *P = 0.0004 (C) Pancreas sections from RIP-gp mice 8 days after receiving peptide vaccine and stained with anti-CD4 or anti-CD8 antibody as shown. (D) Blood glucose measurements from mice vaccinated with peptides, LPS, and anti-CD40 agonistic antibody. Each line represents an individual mouse.

A potential explanation for the lack of diabetes described above is that these vaccination protocols did not induce sufficient expansion of gp-reactive T-cells from naïve precursors in the wild-type repertoire. It was previously shown that the combination of TLR ligands and CD40 agonist in peptide vaccinations can have synergistic effects to promote markedly higher T-cell expansion than either stimulus alone [36]. We therefore combined the administration of gp-derived peptides with LPS and anti-CD40 agonist antibody and assessed T-cell responses. This treatment resulted in a significant increase in gp33–41 tetramer-positive population in the blood compared to mice that received the peptides with LPS alone (Figure 1A and 1B). Furthermore, the combination of LPS and anti-CD40 agonistic antibody in conjunction with peptide vaccination resulted in marked infiltration of pancreatic islets by both CD4^+^ and CD8^+^ cells, which was not observed upon vaccination with peptides and LPS alone (Figure 1C). However, while the combination of the two adjuvants resulted in increased expansion and pancreatic infiltration, no diabetes was observed in mice receiving this treatment (Figure 1D). This indicates that the peptide-based vaccines, while able to prime some T-cell activation and expansion, are unable to induce sufficient CD8 effector responses to result in autoimmunity.

### Transfer of Mature DC Induces Diabetes in RIP-gp Mice

**Figure 2:**
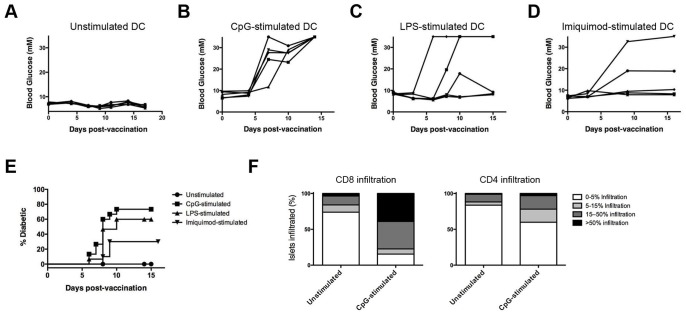
Transfer of mature DC induces diabetes in RIP-gp mice. Bone marrow derived DCs were generated and cultured overnight in media containing (A) no maturation stimulus, (B) CpG ODN 1826 (10 μM), (C) LPS (10 ng/ml), or (D) imiquimod acetate (25 μM), prior to pulsing with gp33–41, gp276–286, and gp61–80 peptides and tail vein infusion to RIP-gp mice at 2×10^6^ DC/mouse. Blood glucose levels were followed after vaccination. Each line represents an individual mouse. (E) Quantification of diabetes incidence following transfer of DC as in (A-D) for 10–20 mice per group. (F) Quantification of the degree of CD8^+^ and CD4^+^ cell infiltration in pancreatic histology 6 days after transfer of unstimulated or CpG-stimulated peptide-pulsed DCs. All results representative of at least 3 independent experiments.

Since DCs have previously been identified as potent initiators of primary T-cell responses [37], we next examined whether diabetes could be induced in RIP-gp mice through the transfer of peptide-pulsed DCs. Bone marrow was harvested from C57BL/6 mice and cultured in LPS-free media containing GM-CSF using a previously established protocol to generate DCs [29]. The transfer of 2×10^6^ DCs pulsed with peptides corresponding to the gp33–41, gp276–286, and gp61–80 epitopes did not result in the induction of diabetes in RIP-gp mice (Figure 2A). However, when DCs were exposed to maturation stimuli, such as CpG ODN 1826 (Figure 2B), LPS (Figure 2C), or imiquimod acetate (Figure 2D) prior to peptide-pulsing and transfer to RIP-gp mice, we observed hyperglycemia between 6 and 10 days post DC-transfer. Induction of diabetes was significantly increased (P<0.02) by maturation of DC with each of the TLR-ligands, however the incidence rate varied with the stimulus used, with CpG-treated DCs inducing significantly greater (P<0.05) incidence of diabetes compared to imiquimod (Figure 2E). Pancreatic histology revealed that the induction of diabetes upon transfer of mature DCs was associated with significantly increased infiltration by CD8^+^ cells (P<0.05 for 5–15% infiltration and >50% infiltration levels) and, to a lesser extent, CD4^+^ cells (Figure 2F). Therefore, while peptide vaccination strategies were unsuccessful at inducing diabetes in the RIP-gp model, DC vaccination promoted effective pancreatic islet destruction in a manner that was dependent on the prior maturation of the transferred DCs.

### DC Transfer Induces CD8 and CD4 Function *in vivo*

**Figure 3:**
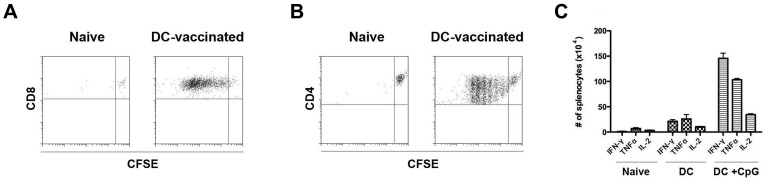
DC transfer induces CD8 and CD4 function *in vivo*. Bone marrow derived DC were generated and cultured overnight in media with or without CpG ODN 1826 (10 μM) prior to peptide-pulsing and transfer via tail vein infusion at 2×10^6^ cells/mouse. (A) 2×10^6^ CFSE-labeled CD8-sorted P14/Thy1.1^+^ splenocytes were transferred to C57BL/6 mice alone or with a separate infusion of peptide-pulsed CpG-stimulated DCs. 3 days later, splenocytes were isolated and flow cytometry was conducted to determine CFSE dilution of transferred Thy1.1^+^CD8^+^ cells. (B) 2×10^6^ CFSE-labeled CD4-sorted Smarta/Thy1.1^+^ splenocytes were transferred to C57BL/6 mice alone or with a separate infusion of peptide-pulsed CpG-stimulated DCs. 3 days later, splenocytes were isolated and flow cytometry was conducted to determine CFSE dilution of transferred Thy1.1^+^CD4^+^ cells. (C) Quantification of gp33–41-specific production of IFN-γ, TNF-α, and IL-2 by CD8^+^ splenocytes in naïve mice and 8 days after vaccination with unstimulated or CpG-stimulated peptide-pulsed DCs. Results show means+/−S.D. Dot plots are gated on Thy1.1^+^ population. All results are representative of 2–3 independent experiments with >3 mice per group.

We next sought to confirm the activation of T-cells by DC transfer *in vivo*. To do so, 2×10^6^ gp33–41 peptide-pulsed, CpG-stimulated DCs were transferred to C57BL/6 mice along with 2×10^6^ CFSE-labeled P14 Thy1.1^+^CD8^+^ splenocytes. Spleens were harvested 3 days later and flow cytometry was conducted to assess division of the transferred congenically marked T-cells. As shown in Figure 3A, Thy1.1^+^ cells underwent CFSE dilution upon cotransfer with peptide-pulsed DC but not when transferred alone, indicating that T-cell division occurred due to the presence of peptide-pulsed DCs *in vivo*. Similarly, co-transfer of 2×10^6^ gp61–80 peptide-pulsed, CpG-stimulated DCs with 2×10^6^ CFSE-labeled Smarta Thy1.1^+^CD4^+^ splenocytes resulted in cell division, indicating that antigen-specific CD4^+^ T-cells could also be engaged by the transfer of peptide-pulsed DCs (Figure 3B). Furthermore, significantly elevated levels of antigen-specific CD8^+^ production of interferon (IFN)-γ, tumor necrosis factor (TNF)-α, and interleukin (IL)-2 were observed upon transfer of TLR-matured peptide-pulsed DCs to non-TCR transgenic C57BL/6 mice compared with mice that had received no vaccine or ‘immature’ peptide-pulsed DCs (Figure 3C). Therefore, these studies demonstrated that peptide-pulsed DCs could activate both CD8^+^ and CD4^+^ T-cells, resulting in antigen-specific proliferation and cytokine production.

**Figure 4:**
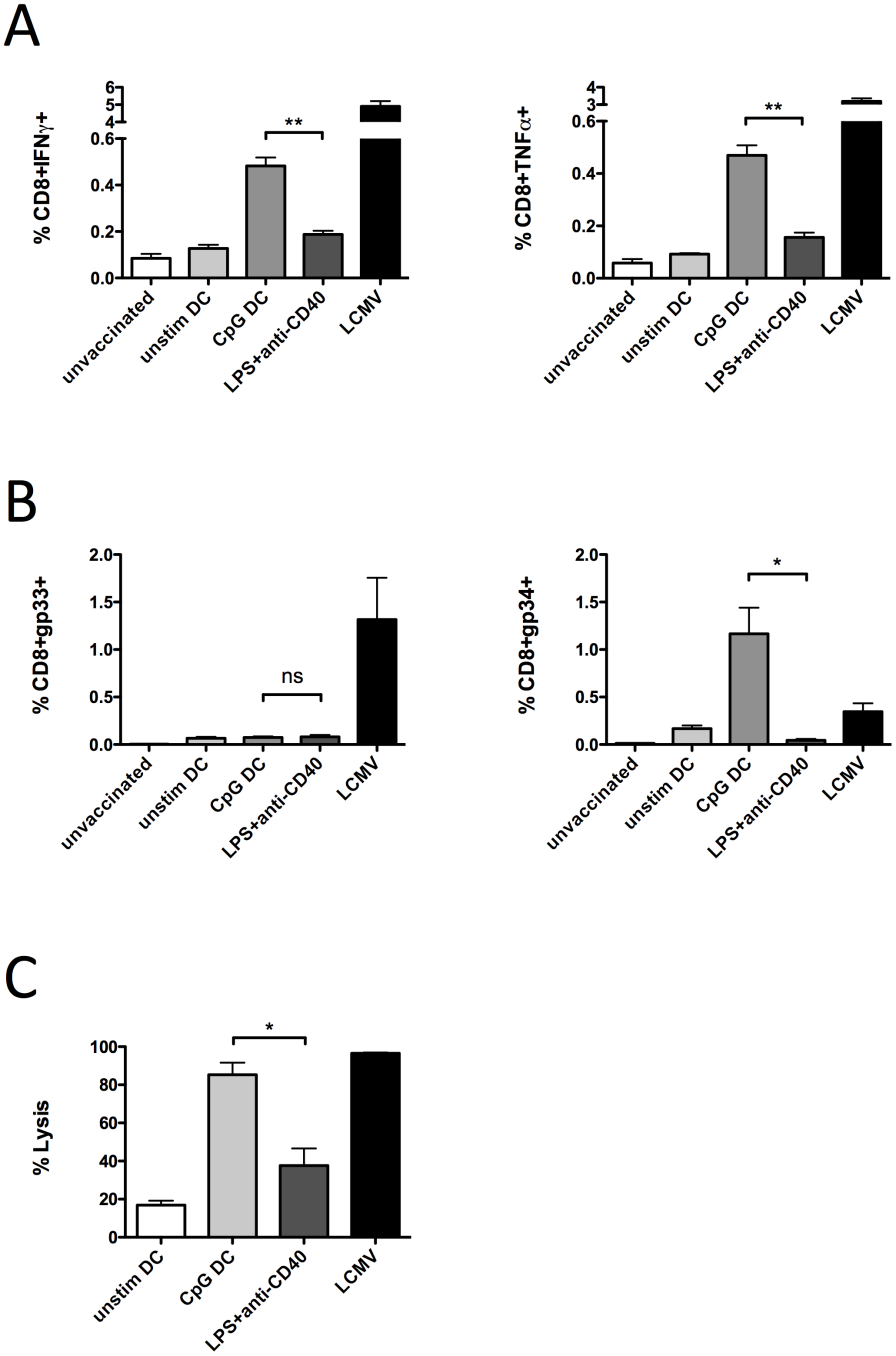
DC transfer promotes enhanced CTL functions compared to peptide plus adjuvant vaccination. Total splenocytes were isolated on day 6 following vaccination with unstimulated DC loaded with peptides gp33–41, gp276–286, and gp61–80 (unstim DC), CpG-matured, peptide-loaded DC (CpG DC), or peptides mixed with LPS and anti-CD40 (LPS+anti-CD40), as well as naive (unvaccinated) and LCMV infected controls. (A) IFN-γ and TNF-α expression in CD8^+^ T-cells were measured following peptide restimulation. (B) Splenocytes were stained with anti-CD8 antibody and gp33–41 or gp-34–41 tetramers. (C) gp33–41 and control AV peptide pulsed and CFSE labeled target cells were transfer into vaccinated mice on day 6 post immunization. 4 hrs later, splenocytes were harvested and examined by flow cytometry for specific lysis of targets. Results show means+/−S.D. from 3 mice per group and are representative of 3–5 independent experiments. **P<0.01; *P<0.05.

To assess whether the enhanced tissue destruction following DC vaccination was associated with enhanced T-cell functions, we compared cytokine responses, antigen-specific T-cell expansion, and target cell lysis between DC vaccinated, peptide vaccinated, and LCMV infected mice. The proportion of CD8^+^ splenocytes producing IFN-γ and TNF-α following DC vaccination was significantly greater (P<0.01) than that produced by vaccination with peptides together with the adjuvants LPS and anti-CD40 (Figure 4A). Comparison of the expansion of gp-specific CD8^+^ T-cell populations restricted by H-2D^b^ (gp33–41-tetramer) versus H-2K^b^ (gp34–41-tetramer) revealed that H-2D^b^ restricted responses were similar between DC and peptide plus adjuvant vaccinated mice (Figure 4B, left panel), while H-2K^b^-restricted T-cell responses were markedly elevated (P<0.05) in mice vaccinated with DCs compared to either peptide plus adjuvant vaccinated or LCMV infected (Figure 4B, right panel). Moreover, CTL responses against gp33–41 labeled target cells were significantly greater (P<0.05) following DC vaccination than after peptide plus adjuvant, and approached the highly efficient CTL activity induced by virus infection (Figure 4C). Vaccination with un-stimulated DCs was not effective at stimulating cytokine responses, antigen-specific CD8^+^ T-cell expansion, or lytic activity. Together these results suggest that DCs carrying self-peptides promote stronger CD8^+^ T-cell responses that allow efficient target destruction, while the combination of LPS plus anti-CD40 adjuvant is unable to sufficiently stimulate CTL functions.

### Pulsing with Multiple Epitopes is Required for Diabetes Induction in RIP-gp Mice

**Figure 5:**
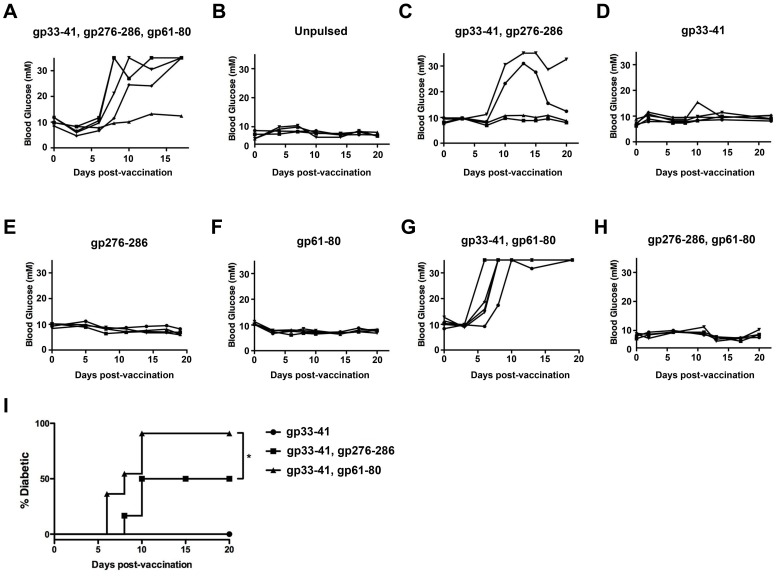
Activation of T-cells specific for at least 2 different peptides is required for diabetes induction. (A-H) Bone marrow derived DCs were matured with 10 μM CpG ODN 1826 and pulsed with the indicated peptides prior to transfer to RIP-gp mice at 2×10^6^ cells/mouse via tail vein infusion. Blood glucose levels were followed on subsequent days. Each line represents an individual mouse. (I) Quantification of diabetes incidence following transfer of CpG-matured DC pulsed with gp33–41 alone, gp33–41 and gp276–286, or gp33–41 and gp61–80. *P<0.02. Results are representative of 10–15 mice per group.

We next sought to examine the requirements for peptide-pulsing of the transferred DCs for the induction of diabetes. As shown in Figures 5A and B, while CpG-stimulated DC that were pulsed with gp33–41, gp276–286 and gp61–80 epitopes induced diabetes approximately a week after transfer, CpG-stimulated DC that were not peptide-pulsed were unable to induce diabetes upon transfer to RIP-gp mice. This finding is consistent with the requirement for antigen-specific activation of T-cells to induce diabetes in this model. We further found that removal of the MHC class II restricted gp61–80 epitope from the peptide cocktail resulted in a lower incidence in diabetes (Figure 5C). Interestingly, however, pulsing with either gp33–41 (Figure 5D), gp276–286 (Figure 5E), or gp61–80 (Figure 5F) alone was insufficient to induce diabetes, indicating that the engagement of multiple TCR specificities was required for effective destruction of the pancreatic islet β-cells. The activation of the immunodominant gp33–41 specific T-cell population was necessary for diabetes induction since DCs pulsed with gp33–41 and gp61–80 induced diabetes (Figure 5G), while transfer of mature DCs that were pulsed with gp276–286 and gp61–80 did not induce diabetes (Figures 5H). Interestingly, the incidence of diabetes by DCs pulsed with the MHC class I and class II binding peptides gp33–41 and gp61–80 was significantly higher than that by DCs pulsed with two MHC class I peptides, gp33–41 and gp276–286, indicating that the engagement of the gp-specific CD4^+^ population was more effective at promoting diabetes than engagement of a second CD8^+^ population (Figure 5I). Combined, these results indicate that diabetes induction in RIP-gp mice through transfer of TLR-stimulated DCs is dependent upon the activation of the endogenous gp33–41 specific CD8^+^ population, in addition to at least one other gp-reactive population.

## Discussion

### Peptide Vaccination versus DC Vaccination

We investigate here the potential for vaccination protocols to break down immunologic ignorance and promote CD8-mediated tissue destruction. Our results clearly demonstrate that DC vaccination strategies are superior to peptide vaccines at inducing antigen-specific destruction of islet cells. Notably, peptide vaccination strategies, while sufficient to induce APC maturation and diabetes in P14/RIP-gp double-transgenic animals, did not promote autoimmune pathology in mice possessing the natural endogenous T-cell repertoire. Tissue destruction was not induced in non-TCR transgenic RIP-gp mice despite the detection of expanded gp-reactive cells in the blood and the infiltration of CD8^+^ and CD4^+^ cells in the pancreatic islets. By maturing endogenous APCs with both LPS and anti-CD40 agonistic antibody, we were able to detect tetramer-positive cells constituting approximately 2% of the CD8^+^ compartment. This is similar to frequencies reported for peptide-induced tumor-specific T-cells in vaccine studies [7], [38]. In clinical studies, measures of successful vaccination often include surrogate markers of immune activation, such as tetramer staining of the blood and histological examination of infiltrates. However, our study and reports by others indicate that these measures may not correlate with actual tumor regression [5].

It is clear that our peptide vaccination strategy does not induce a similar expansion of T-cells to that produced by virus immunization strategies. Of note, we have previously shown that despite the strong T-cell response and memory induced upon LCMV infection, this response is unable to promote a durable anti-tumor response [24], [25]. Therefore, in order to achieve effective cancer immunotherapy, peptide vaccination strategies must be improved to routinely expand a large population of tumor-specific T-cells, and also be combined with approaches that overcome the immunosuppressive factors that are often present in the tumor microenvironment.

We found that gp33–41 peptide-loaded DC vaccination stimulated greater expansion of H-2K^b^-restricted T-cells, a population known to exert potent cytotoxicity following LCMV infection [39] (Kawamura K & Ohashi PS, unpublished observation). Combined with greater IFN-γ and TNF-α production and enhanced cytolytic function, DC vaccination augmented CTL functional responses, compared to vaccination with peptide plus adjuvant, and promoted sufficient tissue destruction to result in diabetes.

It was previously demonstrated that the induction of β-islet cell destruction following LCMV infection of RIP-gp mice was influenced by TNF-α production and MHC class I expression on the target islet cells [40]. Peptide plus TLR-ligand adjuvants were also shown to increase MHC class I expression and IFNα production resulting in target β-islet cell destruction [35]. We did not detected any difference in class I expression on islets cells in RIP-gp mice vaccinated with DCs or peptide plus adjuvant (data not shown). These findings suggest that while expression of MHC class I on the target tissue is absolutely required for target recognition, vaccination-induced CTL expansion and pro-inflammatory cytokine production must reach adequate levels for target tissue destruction to occur.

The LCMV-gp antigen model employed in this study contains high affinity MHC class I and class II epitopes that stimulate potent CD8^+^ and CD4^+^ T-cell responses. A major challenge to tumor-associated antigen (TAA) vaccination is the limited occurrence of high affinity, non-mutated cancer-specific antigen epitopes and cognate high avidity TCR clones in the self-tolerant T-cell repertoire. However, high-affinity mutated TAA epitopes and the occurrence of high avidity TAA-reactive T-cells have been reported, as well as low affinity epitopes capable of stimulating effective anti-tumor responses [41], [42], [43], [44]. The selection of antigen(s) or antigenic peptide(s) that will be most effective for anti-tumor vaccination is, therefore, of critical importance. For weaker TAAs, expansion of reactive T-cells that can effectively target the tumor may require careful dosing or accessory signals [45]. Based on our results, we expect that priming of CTL responses using dendritic cells carrying appropriate TAA-epitopes will have advantages over peptide plus adjuvant(s) at expanding and arming T-cells for targeting TAA-expressing tumors.

Badovinac et al. [46] have demonstrated that DC vaccination can expand antigen-specific T-cells that rapidly acquire memory properties. While we did not examine the memory phenotype of tetramer-positive cells following DC immunization, it is possible that a more rapid generation of memory cells also occurred. However, since the target cell destruction we observed after DC vaccination was very rapid (starting at day 6–10), we believe that an enhanced primary effector CTL response induced by DC immunization was responsible for the effects observed. Comparison of DC vaccination versus peptide immunization in infectious disease protection models will be required to identify whether increased memory cell generation is another potential benefit of DC vaccination.

### The Impact of the Type of Maturation Stimulus on CTL Induction

The above experiments also suggest that different DC maturation stimuli can impact the effectiveness of DC vaccination at eliciting a CTL response, since varying diabetes incidence rates were observed when RIP-gp mice received DCs that had been stimulated with CpG, LPS, or imiquimod. It is possible that stimulation through different pattern recognition receptors (PRRs) leads to expression of different combinations of costimulatory molecules and/or cytokines that can impact the ensuing T-cell response. Accordingly, it has been observed that the engagement of TLR2 or TLR4 on APCs can lead to distinct gene expression profiles and the release of different cocktails of cytokines [47], [48]. Similarly, exposure to LPS, PGN, or zymosan can lead to different levels of IL-10 and IL-12 production by DCs, and the resulting mature DCs can differentially affect Th skewing of CD4^+^ T-cells [49]. In a murine model of autoimmune arthritis that is dependent on endogenous microbial flora for the activation of T-cells, it was shown that deficiency in TLR2 resulted in enhanced pathology correlating with increased IFN-γ producing T-cells and reduced Treg induction, while deficiency in TLR4 resulted in a protective effect that was accompanied by decreased Th17 cells and deficiency in TLR9 had no effect in this system [50]. It is therefore apparent that engagement of different PRRs can impact the induction of an adaptive immune response. The model we present here allows for examination of the impact of maturation stimuli specifically on DCs with regard to their ability to elicit CTL activity against a target organ. As an example, we have shown that IL-12 production by the DCs is essential only in the context of LPS stimulation but not for poly I:C matured DCs [51]. This information may be useful not only for investigating potential immunotherapeutic targets in autoimmune diseases, but may also provide evidence regarding the best stimulus, or combinations of stimuli, to induce anti-tumor CTL responses in the setting of cancer therapy using DC vaccination.

### The Requirement for Engagement of Multiple TCR Specificities

Our studies demonstrated that multiple T-cell specificities must be activated in order to induce effective destruction of the β-cells in the pancreas. In this model, pulsing of DCs with the MHC class I epitope gp33–41 was required for diabetes induction, but gp33–41 peptide presentation alone by matured DCs was insufficient to promote diabetes. This finding may be relevant to clinical trials of DC vaccines for tumors, where some strategies are incorporating the loading of only a single tumor-derived epitope [52], [53]. Our work suggests that the activation of a polyclonal T-cell response may be more effective at eliciting tissue destruction than those that engage a single TCR specificity. Correspondingly, a recent phase II clinical trial found that a multi-peptide vaccine consisting of a 12 peptide mixture provided more robust immunological and clinical responses when compared with a vaccine consisting of a subset of 4 of these peptides [54]. Importantly, the addition of the helper epitope (gp61–80) had a greater impact on the ability of DCs to induce diabetes, compared with the addition of a second MHC class I-restricted epitope (gp276–286). This finding supports the investigation of vaccination strategies that incorporate antigens to prime CD4^+^ cells for more robust tumor regression [55]. A number of MHC class II restricted tumor-derived antigens have been identified in a variety of tumors, including melanoma and breast cancer [56], [57]. Furthermore, recent vaccination strategies have used long synthetic peptides, which are typically approximately 30 amino acids in length, in order to promote peptide internalization, processing, and cross-presentation that would engage CD4^+^ populations [58]. While such efforts might be predicted to enhance the anti-tumor response, a recent clinical trial for a melanoma vaccine found decreased CD8^+^ T-cell responses with the incorporation of helper epitopes [59]. This may reflect the importance of ensuring that the engagement of CD4^+^ T-cells by peptide vaccines is accompanied by proper signals for differentiation, in order to avoid the generation of Th2 or Treg cells that could hinder the cytolytic response [16], [60].

### Perspectives

While advances have been made in tumor antigen-specific expansion of T-cells using vaccination techniques, rates of clinically relevant tumor regression remain low. In order to further evaluate and improve upon cancer vaccination strategies, *in vivo* models are required to assess the degree of tissue cytolysis induced following vaccination. The study presented here demonstrates a model in which vaccination strategies can be compared for their ability to generate T-cells that target the same antigenic target on a tissue, in the absence of tumor immunoevasion mechanisms. Using this system, we found that vaccines using *ex vivo* generated peptide-pulsed DCs are better able to induce tissue destruction compared to vaccine techniques using the same peptides mixed with adjuvant. Correspondingly, a review of clinical trials using peptide vaccines to treat metastatic melanoma estimated an objective tumor regression rate of only 2.7% [5]. The same review reported a tumor regression response rate of 9.5% for DC vaccination strategies for metastatic melanoma [5]. A recent meta-analysis of DC vaccination clinical trials for prostate cancer and renal cell carcinoma also reported objective response rates of 7.7% and 12.7% respectively in these therapy of these tumor types [61]. While these clinical response rates remain low, it is consistent with the work presented here that suggests the transfer of DCs may provide benefits that are not realized through peptide vaccination. Further work will be required to elucidate any such advantages of DC vaccination over peptide vaccination and how they may be exploited to achieve higher clinical response rates. Based on the above work, effective tumor cytolysis will depend upon determination of the ideal combination of stimuli with which the transferred DCs are matured and the activation of a polyclonal population of tumor-specific effector T-cells.
